# Doxycycline Does Not Influence Established Abdominal Aortic Aneurysms in Angiotensin II-Infused Mice

**DOI:** 10.1371/journal.pone.0046411

**Published:** 2012-09-27

**Authors:** Xiaojie Xie, Hong Lu, Jessica J. Moorleghen, Deborah A. Howatt, Debra L. Rateri, Lisa A. Cassis, Alan Daugherty

**Affiliations:** 1 Saha Cardiovascular Research Center, University of Kentucky, Lexington, Kentucky, United States of America; 2 Key Laboratory for Diagnosis and Treatment of Cardiovascular Disease of Zhejiang Province, Department of Cardiology, Second Affiliated Hospital, Zhejiang University College of Medicine, Hangzhou, Zhejiang, People's Republic of China; 3 Graduate Center for Nutritional Sciences, University of Kentucky, Lexington, Kentucky, United States of America; King's College London, University of London, United Kingdom

## Abstract

**Background:**

There is no proven medical approach to attenuating expansion and rupture of abdominal aortic aneurysms (AAAs). One approach that is currently being investigated is the use of doxycycline. Despite being primarily used as an antimicrobial drug, doxycycline has been proposed to function in reducing AAA expansion. Doxycycline is effective in reducing the formation in the most commonly used mouse models of AAAs when administered prior to the initiation of the disease. The purpose of the current study was to determine the effects of doxycycline on established AAAs when it was administered at a dose that produces therapeutic serum concentrations.

**Methods and Results:**

LDL receptor −/− male mice fed a saturated-fat supplemented diet were infused with AngII (1,000 ng/kg/min) via mini-osmotic pumps for 28 days. Upon verification of AAA formation by noninvasive high frequency ultrasonography, mice were stratified based on aortic lumen diameters, and continuously infused with AngII while also administered either vehicle or doxycycline (100 mg/kg/day) in drinking water for 56 days. Administration of doxycycline led to serum drug concentrations of 2.3±0.6 µg/ml. Doxycycline administration had no effect on serum cholesterol concentrations and systolic blood pressures. Doxycycline administration did not prevent progressive aortic dilation as determined by temporal measurements of lumen dimensions using high frequency ultrasound. This lack of effect on AAA regression and progression was confirmed at the termination of the study by *ex vivo* measurements of maximal width of suprarenal aortas and AAA volumes. Also, doxycycline did not reduce AAA rupture. Medial and adventitial remodeling was not overtly changed by doxycycline as determined by immunostaining and histological staining.

**Conclusions:**

Doxycycline administration did not influence AngII-induced AAA progression and aortic rupture when administered to mice with established AAAs.

## Introduction

Abdominal aortic aneurysms (AAAs) represent a progressive disease state with a life-threatening but unpredictable risk for rupture [1]. Currently, no pharmacological intervention has been demonstrated to effectively inhibit the progressive expansion of human AAAs or prevent aortic rupture [2]. One well-recognized characteristic in human AAAs is the increased abundance and activation of matrix metalloproteinases (MMPs) in the diseased aortic tissues [3]–[5].

MMPs are a family of zinc-dependent endopeptidases that are expressed in many cell types. MMPs have been linked to the development of AAAs due to their ability to degrade many extracellular matrix proteins, including elastin and collagen. This associative link of MMPs to AAAs has been enhanced by the detection of many different MMPs in human and experimental aneurysmal tissues, including MMP-1, -2, -3, -7, -8, -9, -12, -13, and MT1-MMP [6]–[11]. A direct role of MMPs on experimental AAAs has been implicated by mouse models with genetic deletion of MMP-2, MMP-9, MMP-12, or MT1-MMP [12]–[15]. For example, deficiency of any of these genes in mice attenuates calcium chloride-induced AAAs [12]–[14], and deficiency of MMP-9 reduces elastase-induced AAAs [15]. However, given the expression of multiple MMPs in aneurysmal tissues and their overlapping substrate selectivity, it has been proposed that an optimal therapeutic strategy in humans would be a drug that broadly inhibits a spectrum of MMPs. Since doxycycline has this property, it has been advocated as a clinically beneficial drug for patients afflicted with AAAs [16].

All three of the commonly used mouse AAA models (elastase- [15], calcium chloride- [12], and angiotensin II (AngII)-induced [17] AAAs) have augmented MMP activation [18]. Furthermore, doxycycline attenuates the formation of experimental AAAs in these mouse models [15], [19], [20]. In these studies, doxycycline was administered prior to application of the initiating event that led to AAA formation. However, in a clinical setting, medical therapy would be initiated following the detection of an established AAA. Consequently, efficacy of potential therapeutic strategies needs to be determined on the effects of progression. AngII infusion for 28 days leads to the formation of AAAs, which have complex pathology [21]–[23]. Continuous infusion beyond 28 days results in progressive AAA expansion and tissue remodeling [24]. Therefore, in the present study, we determined the effects of doxycycline on the progression of established AAAs in mice with prolonged infusion of AngII. Despite achieving serum drug concentrations comparable to those in clinical trials, we were unable to detect an effect of doxycycline on established AAAs.

## Materials and Methods

### Mice and Diet

Male LDL receptor −/− mice on a C57BL/6 background were purchased from The Jackson Laboratory (Stock number 002207, Bar Harbor, Maine, U.S.A.). Mice were housed under barrier conditions and fed normal rodent laboratory diet and water ad libitum. One week prior to mini-osmotic pump implantation, all mice were fed a diet containing milk fat (21% wt/wt) and cholesterol (0.2% wt/wt; TD.88137, Harlan Teklad, Madison, WI, U.S.A.).

### AngII Infusion and Administration of Doxycycline

Mini-osmotic pumps (Alzet Model 2004, Durect Corp, Cupertino, CA, U.S.A.) were implanted subcutaneously to deliver AngII (1,000 ng/kg/min; catalog number A9525, Sigma-Aldrich, St. Louis, MO, U.S.A.) as described previously [17], [25]. Prior to and 24 days after pump implantation, lumen diameters of suprarenal aortas were measured in all mice using a Vevo 660 ultrasound (Visualsonics, Toronto, Ontario, Canada). Mice with established AAAs (≥50% increase of maximal lumen diameter compared to baseline diameter in the suprarenal aorta) were implanted with new mini-osmotic pumps at day 28 and the pumps were replaced at day 56 to permit continuous delivery of AngII for another 56 days. At the 28-day interval, mice were stratified into 2 groups with equivalent sized AAAs. One group was provided with drinking water alone (vehicle), and the other group was administered doxycycline (catalog number D9891, Sigma-Aldrich, St. Louis, MO, U.S.A.). Doxycycline hyclate was dissolved in drinking water at a dose of 100 mg/kg/day and prepared fresh daily. Water bottles containing doxycycline solutions were covered with aluminum foil to protect from light [20].

### Ultrasound Measurement

Lumen diameters of suprarenal aortas were measured using a Vevo 660 ultrasound imaging system in a real-time pattern as described previously [22]. Two-dimensional images (B mode) of short-axis scan were acquired to determine the maximal diameters of suprarenal aortas at selected intervals (weeks 0, 4, 7, and 12 during AngII infusion).

### Systolic Blood Pressure Measurement

Systolic blood pressures were measured on conscious mice using noninvasive tail-cuff systems (BP-2000; Visitech Systems, Inc., Apex, NC, U.S.A.; or CODA 6; Kent Scientific Corp, Torrington, CT, U.S.A.) as described previously [26]. Systolic blood pressures were measured 1 week before mini-osmotic pump implantation to record baseline blood pressures, and repeated on weeks 4, 6, 8, 10, and 12 during AngII infusion.

### Serum Cholesterol and Drug Concentration Measurement

Serum cholesterol concentrations were determined using an enzymatic assay kit (Cholesterol E, catalog number 439-17501, Wako Chemicals USA, Inc., Richmond, VA, U.S.A.) as described previously [27]. Serum doxycycline concentrations were measured using reverse-phase high performance liquid chromatography with UV detection as described previously [28].

### AAA Quantification

During termination, aortas were excised after pressure perfusion at 100 mmHg with 10% neutrally buffered formalin and injected with 3% (wt/vol) agarose to maintain patency. AAAs were quantified by measuring ex vivo maximal diameter of suprarenal aortas using Image-Pro Plus software (Media Cybernetics, Bethesda, MD, U.S.A.) [29]. Volume of each AAA was measured using the three-dimensional imaging function of the Vevo 660 ultrasound.

### Histological Staining and Immunostaining

Abdominal aortas containing AAAs were serially cross-sectioned (10 µm thick/section) from the proximal to the distal as described previously [21], [24]. Collagen content was determined with picrosirius red staining. Immunostaining was performed to identify macrophages and smooth muscle cells as described previously [30]. The following primary antibodies were used: rabbit antisera against mouse macrophages (Catalog number AIAD31240, Accurate Chemical & Scientific Corp, Westbury, NY, U.S.A.) and rabbit polyclonal antibody against alpha smooth muscle actin (catalog number ab5694, Abcam, Cambridge, MA, U.S.A.).

### Statistical Analysis

Data are presented as means ± standard error of means (SEM). SigmaPlot version 12 (Systat Software Inc., San Jose, CA, USA) was used for statistical analyses. Two-group comparisons were performed using Student's *t* test for normally and equally distributed data and Mann-Whitney Rank Sum analysis for data having failed either normality or equal variance test. Weekly body weight, systolic blood pressure, and aortic diameters measured at selected time points with ultrasound were analyzed using two way repeated measures ANOVA. Aortic rupture rate during prolonged AngII infusion (Days 28–84) was compared between the two groups (Vehicle versus Doxycycline) using LogRank survival analysis. A P<0.05 was considered to be significant.

### Ethics Statement

All mouse studies were performed with approval of the University of Kentucky Institutional Animal Care and Use Committee (IACUC protocol number: 2006-0009).

## Results

### Characteristics of Study Mice

**Figure 1:**
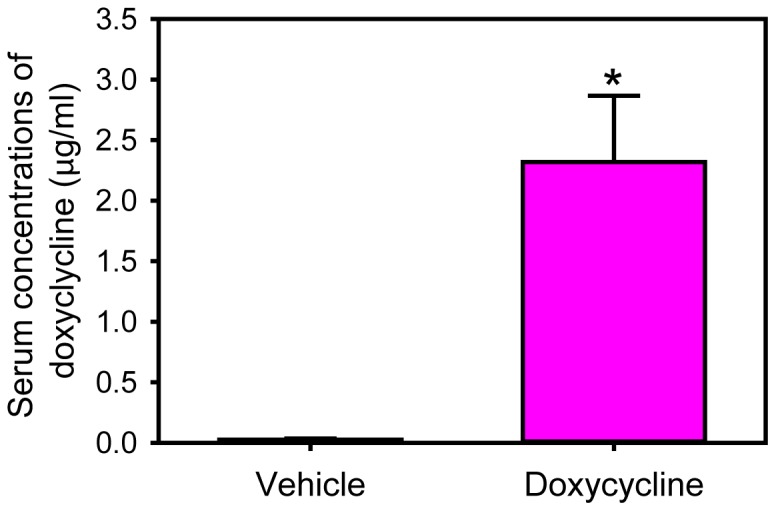
Measurements of serum doxycycline concentrations. Serum doxycycline concentrations were measured using reverse-phase high performance liquid chromatography. Histobars represent means, and error bars represent SEM (N = 9 in vehicle group and N = 10 in doxycycline group). * denotes P<0.001 by Mann-Whitney Rank Sum analysis.

**Figure 2:**
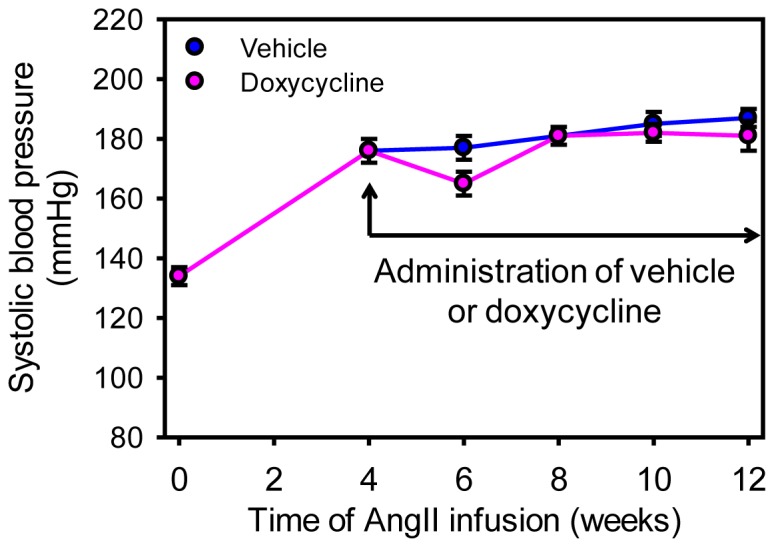
Doxycycline did not change AngII-induced increases of systolic blood pressures. Systolic blood pressures were measured using a tail-cuff system prior to pump implantation (week 0) and at weeks 4, 6, 8, 10, and 12 during AngII infusion. Vehicle or doxycycline administration in drinking water started after 4 weeks of AngII infusion. Circles represent mean values at each time point and bars represent SEM. N = 9 and 10 for vehicle and doxycycline groups, respectively. Statistical analysis was performed using two way repeated measures ANOVA. P<0.001 for the comparisons of the AngII infusion interval. P = 0.50 for the comparisons between the two groups.

Forty-one male LDL receptor −/− mice were infused with AngII (1,000 ng/kg/min) for 28 days before these mice were administered either the vehicle or doxycycline. During the 28-day infusion with AngII, 9 mice (22%) died of aortic rupture. AAA formation was confirmed in 25 of the remaining 32 mice (78%) by ultrasound at day 24 of AngII infusion. Subsequently, mice with established AAAs (N = 25) were stratified to receive vehicle (N = 11) or doxycycline (N = 14) 28 days after AngII infusion. Doxycycline given in drinking water was well tolerated as determined by daily observation and body weight measurements on a weekly base. Oral administration of doxycycline at a dose of 100 mg/kg/day led to serum drug concentrations of 2.3±0.6 µg/ml as measured using reverse-phase high performance liquid chromatography (Figure 1). In mice infused with AngII for a prolonged interval, doxycycline administration had no effects on body weight, systolic blood pressure (Figure 2), and serum cholesterol concentrations (vehicle versus doxycycline: 1429±61 and 1227±97, respectively; P>0.05).

### Doxycycline Did Not Regress or Prevent the Progression of AngII-induced AAAs

**Figure 3:**
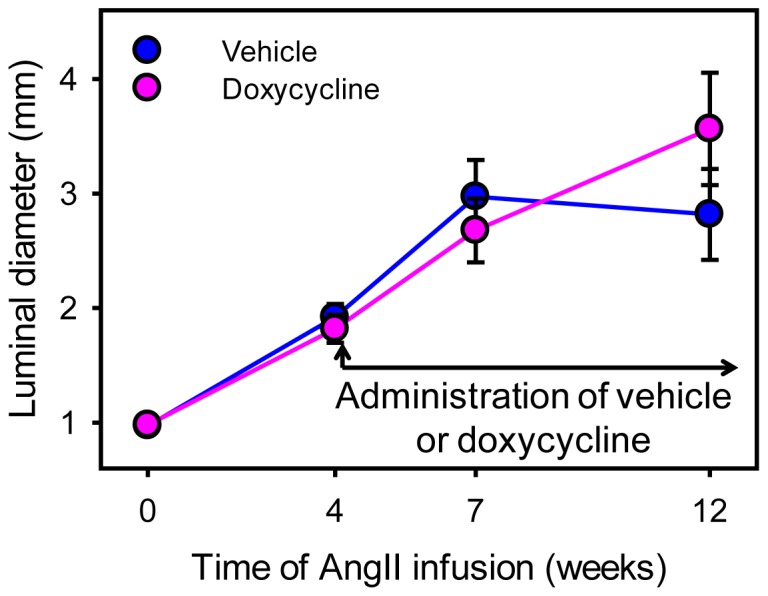
Doxycycline did not reduce lumen expansion rates of AngII-induced AAAs. Maximal luminal diameters of suprarenal aortas were measured by ultrasonography prior to (week 0) and at weeks 4, 7, and 12 during AngII infusion. Circles represent mean values of each time point and bars represent SEM. N = 9 in vehicle group and N = 10 in doxycycline group. Statistical analysis was performed using two way repeated measures ANOVA. P<0.001 for the comparisons of the AngII infusion interval. P = 0.71 for the comparisons between the two groups.

**Figure 4:**
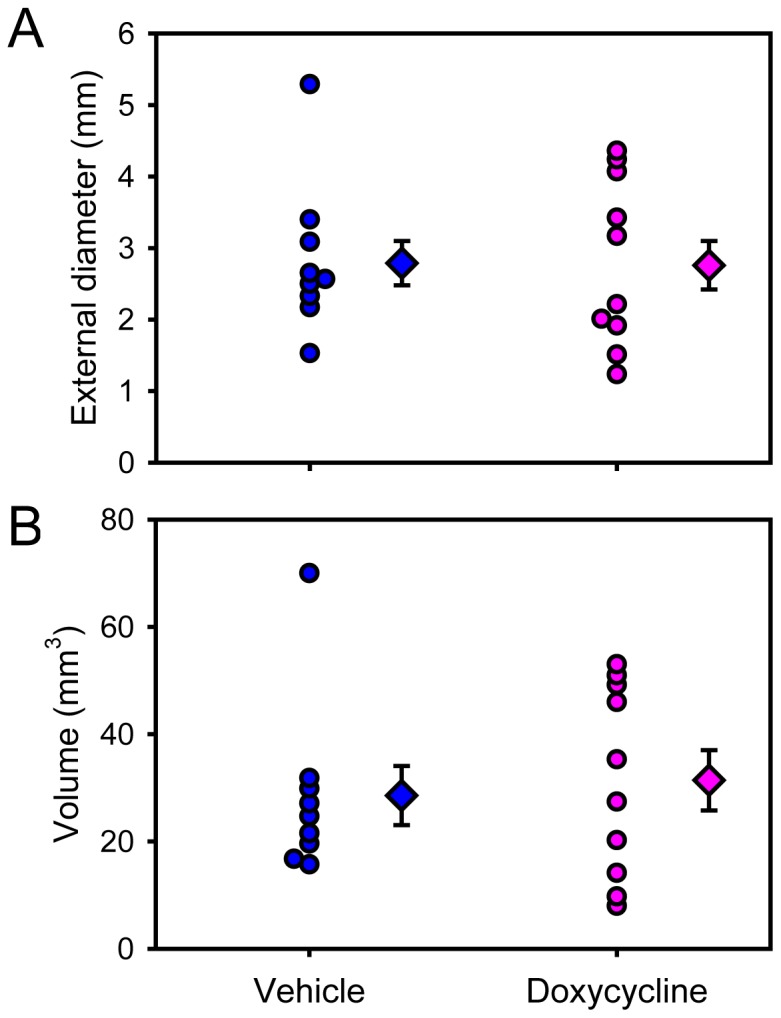
Doxycycline did not change aortic widths or lumen volumes of AngII-induced AAAs. (**A**) Maximal external widths of suprarenal aortas were measured using Image-Pro Plus software. (**B**) Volumes of suprarenal aortas were measured on three-dimensional reconstructed images of ex vivo AAAs. Circles represent the values of individual mice (N = 9 in vehicle group and N = 10 in doxycycline group), diamonds represent means, and bars are SEM. Student's *t* test was performed to compare the two groups for both aortic widths and lumen volumes. P = 0.97 and 0.72, respectively.

**Figure 5:**
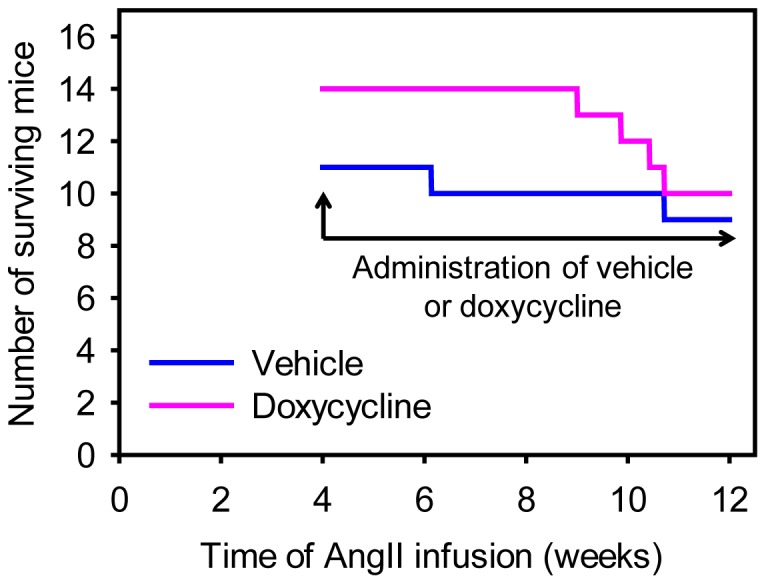
Doxycycline did not influence deaths caused by aortic rupture. Mouse number after excluding dead mice due to aortic rupture was recorded from the beginning of doxycycline administration (day 28 during AngII infusion) to the termination of study mice (day 84 during AngII infusion). Each line represents number of live mice on each day during AngII infusion. Aortic rupture rate between the two groups was analyzed using LogRank survival analysis. P = 0.65.

Protracted AngII infusion led to progressive luminal expansion of suprarenal aortas as monitored by ultrasonography (Figure 3), which was consistent with our previous report [24]. Doxycycline did not attenuate the expansion rate of suprarenal aortic diameters measured temporally with ultrasound. This lack of effect as determined by the noninvasive imaging was confirmed after termination by ex vivo maximal width of suprarenal aortas (Figure 4A). Furthermore, three-dimensional AAA imaging reconstruction demonstrated that doxycycline did not change AAA volume (Figure 4B). In addition, doxycycline did not influence the incidence of death caused by aortic rupture as determined by necropsy (Figure 5).

### Doxycycline Did Not Change Cellular and Extracellular Characteristics of AAA Tissues

**Figure 6:**
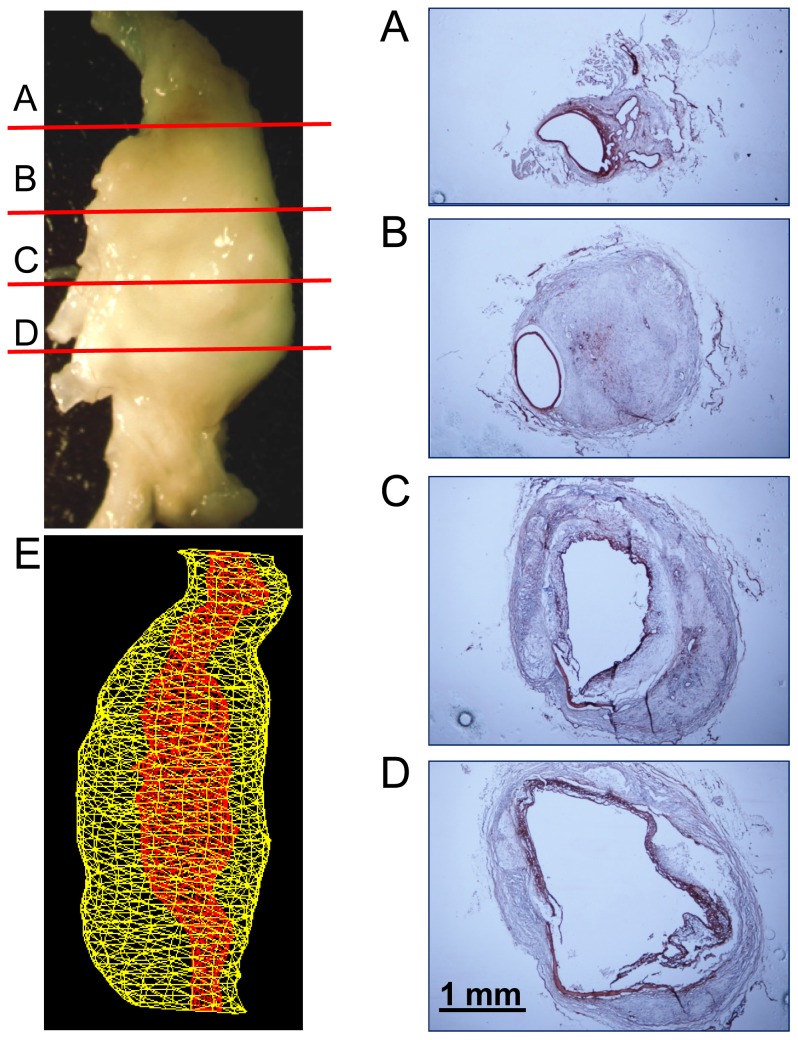
Examples of AngII-induced AAAs in an LDL receptor −/− mouse administered vehicle. (**A**)–(**D**) Heterogenous pathological features of the AAA from the proximal to the distal as shown by immunostaining of alpha smooth muscle actin. (**E**) Volume of the AAA was measured using the 3-dimensional imaging function of a Vevo 660 ultrasound.

**Figure 7:**
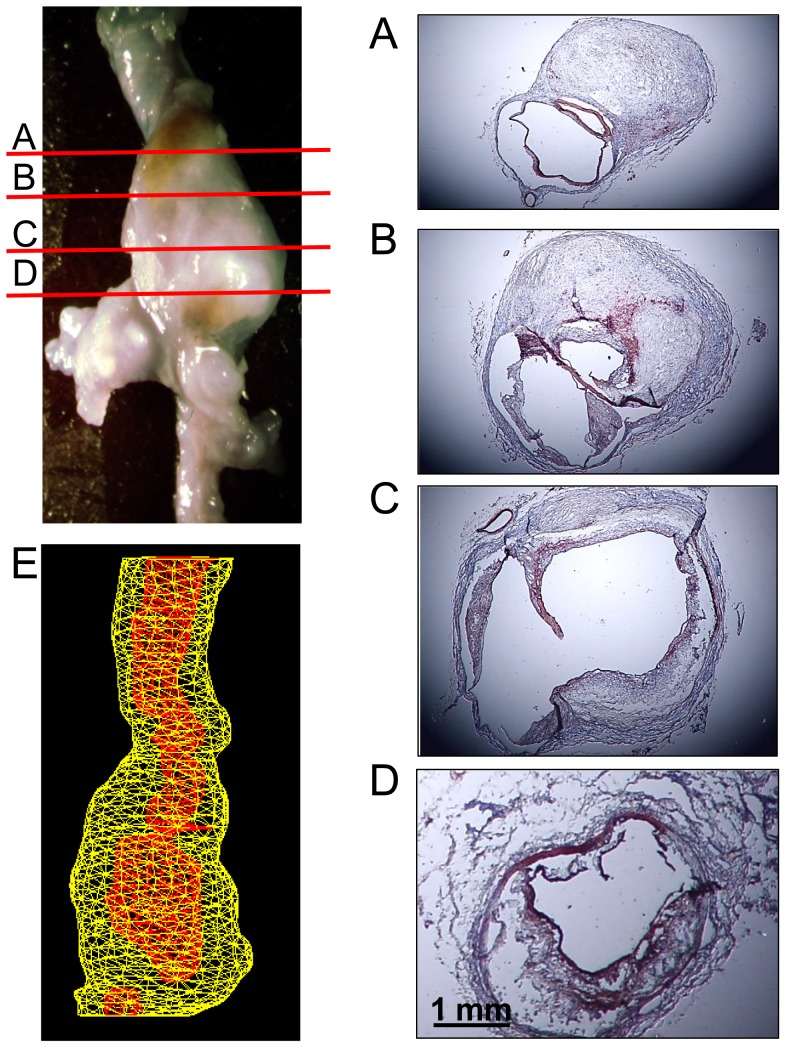
Examples of AngII-induced AAAs in an LDL receptor −/− mice administered doxycycline. (**A**)–(**D**) Heterogenous pathological features of the AAA from the proximal to the distal as shown by immunostaining of alpha smooth muscle actin. (**E**) Volume of the AAA was measured using the 3-dimensional imaging function of a Vevo 660 ultrasound.

Pathologies of AngII-induced AAAs in advanced stages are highly heterogeneous, exhibiting complex features and differing markedly along the length of a single aneurysm [21], [24]. To determine whether broad inhibition of MMPs by doxycycline influenced pathological characteristics, AAAs were serially cross-sectioned throughout the region of aortic expansion. Consistent with our previous study [24], prolonged AngII infusion resulted in transmedial rupture that occurred predominantly at the left anterior aspect of the suprarenal aortic region. Profound neovascularization was present in adventitia as demonstrated by positive smooth muscle alpha-actin staining, particularly surrounding the regions of medial rupture. Pronounced accumulation of macrophages was detected in both aortic aneurysmal tissues and the adventitia surrounding the AAA. Cellular elements and collagen deposition were markedly heterogeneous even within a single aneurysm. There was no overt difference in the cellular and matrix contents in AAAs between mice administered vehicle and doxycycline (Figures 6 and 7).

## Discussion

Doxycycline suppresses formation of experimental AAAs as demonstrated in both rat and mouse models [3], [15], [19], [20], [31]–[36]. In all these studies, doxycycline was administered during the initiative phase of AAAs. In contrast as shown in the present study, doxycycline did not influence established AAAs in AngII-infused hypercholesterolemic mice, although effective serum concentrations of the drug were achieved. Additionally, doxycycline did not change either aortic rupture rate or pathological characteristics of AngII-induced AAAs.

While doxycycline is a widely used antibiotic, it is also a well-recognized broad-spectrum inhibitor of MMPs. It has been reported that doxycycline reduces experimental AAAs via inhibiting MMP activation [15], [19], [31], [33], [34], [36]. In our [20] and a recently reported [36] studies, doxycycline at a dose of 30 mg/kg/day was provided in drinking water during subcutaneous infusion of AngII for 4 weeks. This dose of doxycycline profoundly reduced AngII-induced AAAs and the rate of aortic rupture [20], [36]. In the present study, we administered doxycycline at a dose of 100 mg/kg/day in drinking water in order to achieve maximal inhibitory effects on MMP activation [19]. This dose has been demonstrated to efficiently inhibit MMP activation in AAA tissues from humans and animal models [19], [35]. In the present study, this dose and the mode of administration resulted in a mean serum doxycycline concentration of 2.3 µg/ml that is within the effective range to inhibit MMP activity [19], [35]. Despite achieving effective serum concentrations, doxycycline had no effect on established AAAs in AngII-infused mice as measured by several in vivo and ex vivo modalities. During AngII infusion and doxycycline administration, luminal expansion of suprarenal aortas was monitored using ultrasonography. We observed equivalently progressive luminal dilation in both the vehicle and doxycycline administered mice. In vivo ultrasonic measurements prior to termination were confirmed to be comparable with the ex vivo aortic width measurements of suprarenal aortas. We also measured volume of each AAA and obtained similar results as the other measurements between the two groups. These different approaches provided compelling evidence that doxycycline did not influence the size of established AAAs in AngII-infused hypercholesterolemic mice. Aortic rupture, the devastating consequence of AAAs that occurred in nearly any stage during AAA progression, was not significantly influenced by administration of doxycycline during the protracted AngII infusion.

AAA diameter is the most commonly used parameter to monitor the progression of AAAs and is also used to represent the risk for aortic rupture in patients [2]. A growing body of evidence provides mechanistic insights that progression of AAAs and the potential to rupture may be driven by complex pathological features, rather than being simply associated with increases of aneurysmal size. Hence, in addition to the quantification of AAA size by multiple processes, we also characterized AAAs with histological and immunological stainings. In agreement with our recent report [24], prolonged infusion of AngII resulted in progressively disorganized extracellular matrix, transmural tissue remodeling, and extensive macrophage accumulation in both aneurysms and the adventitia surrounding AAAs. We were unable to distinguish any difference in the pathology of aneurysms in aortic tissues retrieved from vehicle versus doxycycline administered mice. In addition to macrophage infiltration, neutrophils (as determined by immunostaining of myeloperoxidase) and cytotoxic (CD8+) T lymphocytes are abundant in human AAA tissue [37]. The numbers of these cell types were equivalently reduced by 2 weeks of administering doxycycline at doses of 50–300 mg/day [37]. In contrast to these human studies, we have not been able to detect neutrophils in AngII-infused AAAs in mice, although both T and B lymphocytes are present [17], [21]. Their functional role is unclear since deficiency of both T and B lymphocytes has no effect on the formation of AAAs during 28 days of AngII infusion [38]. It remains to be determined whether total lymphocyte deficiency has an effect on progression of established AAAs.

Although many studies have demonstrated that some drugs and genetic deletions ameliorate the initiation of AAAs [39], only JNK inhibition has been reported to cause regression of established experimental AAAs [40]. An early event during the initiation of AngII-induced AAAs is transmural medial rupture that leads to large lumen expansions [21], [22]. Therefore, the initial process requires the destruction of extracellular matrix to enable AAA formation. Doxycycline prevents the development of AngII-induced AAAs [20], [36], but does not influence AAA size and rupture in mice with established AAAs. MMP activity might be compatible with the rapid destruction of extracelluar matrix in the formative stage and explain the previous demonstration of doxycycline reducing AAAs in this stage. Following the initiation of AngII-induced AAAs, continuous infusion leads to slower and progressive lumen expansion that is accompanied by tissue remodeling. This phase is characterized by complex changes in extracellular matrix [21], [24]. These temporal changes in tissue characteristics are consistent with the mechanisms in the progression phase, but differ dramatically from the initiative phase. Consequently, there is likely to be disparities in drug effects at different phases of AAAs. Considering the deleterious damage and complex remodeling of the aortic wall, it is also possible that doxycycline may not be effectively delivered to the diseased aorta.

The effects of doxycycline on AAAs have previously been reported in humans. A small clinical trial involving 32 patients received either placebo or doxycycline (150 mg daily) for 3 months and AAA diameter was followed for 18 months [41]. Although interim analyses observed reduction of AAA expansion in patients that received doxycycline, there was no effect at the 18 month end point of the trial. Another small trial of 36 patients also failed to demonstrate that doxycycline (200 mg daily) influenced AAA diameter over a 6-month interval [42]. The shortcomings, such as small number of patients and too short period of the drug administration, of these pilot studies have been addressed in 2 ongoing clinical trials (NCT00538967and [16]). Although these pitfalls may attribute to the negative results, it is also possible, as inferred by the present study, that inhibition of MMPs via administering doxycycline may not influence established AAAs.

In conclusion, although doxycycline prevents the initiation of AngII-induced AAAs, it does not retard or obviate the progression of AAAs, or prevent rupture once AAAs have established. While MMPs may play a divergent role in the initiative and progressive stages of AAAs, further studies are necessary to define the molecular mechanisms that are responsible for the progression of AAAs before effective therapeutic strategies may be explored for established AAAs.

## References

[1] Powell JT, Sweeting MJ, Brown LC, Gotensparre SM, Fowkes FG, et al (2011) Systematic review and meta-analysis of growth rates of small abdominal aortic aneurysms. Br J Surg 98: 609–618. 21412998 10.1002/bjs.7465

[2] Baxter BT, Terrin MC, Dalman RL (2008) Medical management of small abdominal aortic aneurysms. Circulation 117: 1883–1889. 18391122 10.1161/CIRCULATIONAHA.107.735274 PMC4148043

[3] Petrinec D, Liao S, Holmes DR, Reilly JM, Parks WC, et al (1996) Doxycycline inhibition of aneurysmal degeneration in an elastase-induced rat model of abdominal aortic aneurysm: preservation of aortic elastin associated with suppressed production of 92 kD gelatinase. J Vasc Surg 23: 336–346. 8637112 10.1016/s0741-5214(96)70279-3

[4] Choke E, Cockerill G, Wilson WR, Sayed S, Dawson J, et al (2005) A review of biological factors implicated in abdominal aortic aneurysm rupture. Eur J Vasc Endovasc Surg 30: 227–244. 15893484 10.1016/j.ejvs.2005.03.009

[5] Pearce WH, Shively VP (2006) Abdominal aortic aneurysm as a complex multifactorial disease: interactions of polymorphisms of inflammatory genes, features of autoimmunity, and current status of MMPs. Ann NY Acad Sci 1085: 117–132. 17182928 10.1196/annals.1383.025

[6] Newman KM, Jean-Claude J, Li H, Scholes JV, Ogata Y, et al (1994) Cellular localization of matrix metalloproteinases in the abdominal aortic aneurysm wall. J Vasc Surg 20: 814–820. 7526009 10.1016/s0741-5214(94)70169-5

[7] Freestone T, Turner RJ, Coady A, Higman DJ, Greenhalgh RM, et al (1995) Inflammation and matrix metalloproteinases in the enlarging abdominal aortic aneurysm. Arterioscler Thromb Vasc Biol 15: 1145–1151. 7627708 10.1161/01.atv.15.8.1145

[8] Fontaine V, Jacob MP, Houard X, Rossignol P, Plissonnier D, et al (2002) Involvement of the mural thrombus as a site of protease release and activation in human aortic aneurysms. Am J Pathol 161: 1701–1710. 12414517 10.1016/S0002-9440(10)64447-1 PMC1850780

[9] Wilson WR, Anderton M, Schwalbe EC, Jones JL, Furness PN, et al (2006) Matrix metalloproteinase-8 and -9 are increased at the site of abdominal aortic aneurysm rupture. Circulation 113: 438–445. 16432074 10.1161/CIRCULATIONAHA.105.551572

[10] Curci JA, Liao SX, Huffman MD, Shapiro SD, Thompson RW (1998) Expression and localization of macrophage elastase (Matrix metalloproteinase-12) in abdominal aortic aneurysms. J Clin Invest 102: 1900–1910. 9835614 10.1172/JCI2182 PMC509141

[11] Mao D, Lee JK, VanVickle SJ, Thompson RW (1999) Expression of collagenase-3 (MMP-13) in human abdominal aortic aneurysms and vascular smooth muscle cells in culture. Biochem Biophys Res Commun 261: 904–910. 10441523 10.1006/bbrc.1999.1142

[12] Longo GM, Xiong W, Greiner TC, Zhao Y, Fiotti N, et al (2002) Matrix metalloproteinases 2 and 9 work in concert to produce aortic aneurysms. J Clin Invest 110: 625–632. 12208863 10.1172/JCI15334 PMC151106

[13] Longo GM, Buda SJ, Fiotta N, Xiong W, Griener T, et al (2005) MMP-12 has a role in abdominal aortic aneurysms in mice. Surgery 137: 457–462. 15800495 10.1016/j.surg.2004.12.004

[14] Xiong W, Knispel R, MacTaggart J, Greiner TC, Weiss SJ, et al (2009) Membrane-type 1 matrix metalloproteinase regulates macrophage-dependent elastolytic activity and aneurysm formation in vivo. J Biol Chem 284: 1765–1771. 19010778 10.1074/jbc.M806239200 PMC2615502

[15] Pyo R, Lee JK, Shipley JM, Curci JA, Mao D, et al (2000) Targeted gene disruption of matrix metalloproteinase-9 (gelatinase B) suppresses development of experimental abdominal aortic aneurysms. J Clin Invest 105: 1641–1649. 10841523 10.1172/JCI8931 PMC300851

[16] Thompson RW, Baxter BT (1999) MMP inhibition in abdominal aortic aneurysms. Rationale for a prospective randomized clinical trial. Ann NY Acad Sci 878: 159–178. 10415728 10.1111/j.1749-6632.1999.tb07682.x

[17] Daugherty A, Manning MW, Cassis LA (2000) Angiotensin II promotes atherosclerotic lesions and aneurysms in apolipoprotein E-deficient mice. J Clin Invest 105: 1605–1612. 10841519 10.1172/JCI7818 PMC300846

[18] Daugherty A, Cassis LA (2004) Mouse models of abdominal aortic aneurysms. Arterioscler Thromb Vasc Biol 24: 429–434. 14739119 10.1161/01.ATV.0000118013.72016.ea

[19] Prall AK, Longo GM, Mayhan WG, Waltke EA, Fleckten B, et al (2002) Doxycycline in patients with abdominal aortic aneurysms and in mice: comparison of serum levels and effect on aneurysm growth in mice. J Vasc Surg 35: 923–939. 12021708 10.1067/mva.2002.123757

[20] Manning MW, Cassis LA, Daugherty A (2003) Differential effects of doxycycline, a broad-spectrum matrix metalloproteinase inhibitor, on angiotensin II-induced atherosclerosis and abdominal aortic aneurysms. Arterioscler Thromb Vasc Biol 23: 483–488. 12615694 10.1161/01.ATV.0000058404.92759.32

[21] Saraff K, Babamusta F, Cassis LA, Daugherty A (2003) Aortic dissection precedes formation of aneurysms and atherosclerosis in angiotensin II-infused, apolipoprotein E-deficient mice. Arterioscler Thromb Vasc Biol 23: 1621–1626. 12855482 10.1161/01.ATV.0000085631.76095.64

[22] Barisione C, Charnigo R, Howatt DA, Moorleghen JJ, Rateri DL, et al (2006) Rapid dilation of the abdominal aorta during infusion of angiotensin II detected by noninvasive high-frequency ultrasonography. J Vasc Surg 44: 372–376. 16890871 10.1016/j.jvs.2006.04.047

[23] Daugherty A, Cassis LA, Lu H (2011) Complex pathologies of angiotensin II-induced abdominal aortic aneurysms. J Zhejiang Univ Sci B 12: 624–628. 21796801 10.1631/jzus.B1101002 PMC3150714

[24] Rateri DL, Howatt DA, Moorleghen JJ, Charnigo R, Cassis LA, et al (2011) Prolonged infusion of angiotensin II in apoE−/− mice promotes macrophage recruitment with continued expansion of abdominal aortic aneurysms. Am J Pathol 179: 1542–1548. 21763672 10.1016/j.ajpath.2011.05.049 PMC3157213

[25] Daugherty A, Cassis L (1999) Chronic angiotensin II infusion promotes atherogenesis in low density lipoprotein receptor −/− mice. Ann NY Acad Sci 892: 108–118. 10842656 10.1111/j.1749-6632.1999.tb07789.x

[26] Daugherty A, Rateri D, Lu H, Balakrishnan A (2009) Measuring blood pressure in mice using volume pressure recording, a tail-cuff method. J Vis Exp 1291. 10.3791/1291 PMC2794298 19488026

[27] Daugherty A, Rateri DL (1994) Presence of LDL receptor-related protein/alpha 2-macroglobulin receptors in macrophages of atherosclerotic lesions from cholesterol- fed New Zealand and heterozygous Watanabe heritable hyperlipidemic rabbits. Arterioscler Thromb 14: 2017–2024. 7526898 10.1161/01.atv.14.12.2017

[28] Axisa B, Naylor AR, Bell PR, Thompson MM (2000) Simple and reliable method of doxycycline determination in human plasma and biological tissues. J Chromatogr B Biomed Sci Appl 744: 359–365. 10993525 10.1016/s0378-4347(00)00261-9

[29] Wang YX, Cassis LA, Daugherty A (2006) Angiotensin II-induced abdominal aortic aneurysms. A Handbook of Mouse Models for Cardiovascular Disease Ed. Q. Xu, John Wiley & Sons: 125–136.

[30] Lu H, Rateri DL, Daugherty A (2007) Immunostaining of mouse atherosclerosis lesions. Methods Mol Med 139: 77–94. 18287665 10.1007/978-1-59745-571-8_4

[31] Petrinec D, Holmes DR, Liao S, Golub LM, Thompson RW (1996) Suppression of experimental aneurysmal degeneration with chemically modified tetracycline derivatives. Ann NY Acad Sci 800: 263–265. 8959009 10.1111/j.1749-6632.1996.tb33326.x

[32] Curci JA, Petrinec D, Liao S, Golub LM, Thompson RW (1998) Pharmacologic suppression of experimental abdominal aortic aneurysms: a comparison of doxycycline and four chemically modified tetracyclines. J Vasc Surg 28: 1082–1093. 9845660 10.1016/s0741-5214(98)70035-7

[33] Kaito K, Urayama H, Watanabe G (2003) Doxycycline treatment in a model of early abdominal aortic aneurysm. Surg Today 33: 426–433. 12768368 10.1007/s10595-002-2513-0

[34] Sho E, Chu J, Sho M, Fernandes B, Judd D, et al (2004) Continuous periaortic infusion improves doxycycline efficacy in experimental aortic aneurysms. J Vasc Surg 39: 1312–1321. 15192574 10.1016/j.jvs.2004.01.036

[35] Bartoli MA, Parodi FE, Chu J, Pagano MB, Mao D, et al (2006) Localized administration of doxycycline suppresses aortic dilatation in an experimental mouse model of abdominal aortic aneurysm. Ann Vasc Surg 20: 228–236. 16572291 10.1007/s10016-006-9017-z

[36] Turner GH, Olzinski AR, Bernard RE, Aravindhan K, Karr HW, et al (2008) In vivo serial assessment of aortic aneurysm formation in apolipoprotein E-deficient mice via MRI. Circ Cardiovasc Imaging 1: 220–226. 19808546 10.1161/CIRCIMAGING.108.787358

[37] Lindeman JH, Abdul-Hussien H, van Bockel JH, Wolterbeek R, Kleemann R (2009) Clinical trial of doxycycline for matrix metalloproteinase-9 inhibition in patients with an abdominal aneurysm: doxycycline selectively depletes aortic wall neutrophils and cytotoxic T cells. Circulation 119: 2209–2216. 19364980 10.1161/CIRCULATIONAHA.108.806505

[38] Uchida HA, Kristo F, Rateri DL, Lu H, Charnigo R, et al (2010) Total lymphocyte deficiency attenuates AngII-induced atherosclerosis in males but not abdominal aortic aneurysms in apoE deficient mice. Atherosclerosis 211: 399–403. 20362292 10.1016/j.atherosclerosis.2010.02.034 PMC2900415

[39] Golledge J, Muller J, Daugherty A, Norman P (2006) Abdominal aortic aneurysm. Pathogenesis and implications for management. Arterioscler Thromb Vasc Biol 26: 2605–2613. 16973970 10.1161/01.ATV.0000245819.32762.cb

[40] Yoshimura K, Aoki H, Ikeda Y, Fujii K, Akiyama N, et al (2005) Regression of abdominal aortic aneurysm by inhibition of c-Jun N-terminal kinase. Nat Med 11: 1330–1338. 16311603 10.1038/nm1335

[41] Mosorin M, Juvonen J, Biancari F, Satta J, Surcel HM, et al (2001) Use of doxycycline to decrease the growth rate of abdominal aortic aneurysms: A randomized, double-blind, placebo-controlled pilot study. J Vasc Surg 34: 606–610. 11668312 10.1067/mva.2001.117891

[42] Baxter BT, Pearce WH, Waltke EA, Littooy FN, Hallett JW Jr, et al (2002) Prolonged administration of doxycycline in patients with small asymptomatic abdominal aortic aneurysms: report of a prospective (Phase II) multicenter study. J Vasc Surg 36: 1–12. 12096249 10.1067/mva.2002.125018

